# KNL1 is a prognostic and diagnostic biomarker related to immune infiltration in patients with uterine corpus endometrial carcinoma

**DOI:** 10.3389/fonc.2023.1090779

**Published:** 2023-01-27

**Authors:** Kang He, Jingze Li, Xuemiao Huang, Weixin Zhao, Kai Wang, Taiwei Wang, Junyu Chen, Zeyu Wang, Jiang Yi, Shuhua Zhao, Lijing Zhao

**Affiliations:** ^1^ Department of Rehabilitation, School of Nursing, Jilin University, Changchun, China; ^2^ The Department of Obstetrics and Gynecology, The Second Hospital of Jilin University, Changchun, Jilin, China; ^3^ Department of Rehabilitation, The Second Hospital of Jilin University, Changchun, Jilin, China

**Keywords:** KNL1, uterine corpus endometrial carcinoma, bioinformatics, prognosis, biomarker

## Abstract

**Background:**

The incidence and mortality of uterine corpus endometrial carcinoma (UCEC) are increasing yearly. There is currently no screening test for UCEC, and progress in its treatment is limited. It is important to identify new biomarkers for screening, diagnosing and predicting the outcomes of UCEC. A large number of previous studies have proven that KNL1 is crucial in the development of lung cancer, colorectal cancer and cervical cancer, but there is a lack of studies about the role of KNL1 in the development of UCEC.

**Methods:**

The mRNA and protein expression data of KNL1 in The Cancer Genome Atlas (TCGA), Gene Expression Omnibus (GEO) and UALCAN databases and related clinical data were used to analyze the expression differences and clinical correlations of KNL1 in UCEC. A total of 108 clinical samples were collected, and the results of bioinformatics analysis were verified by immunohistochemistry. KNL1 and its related differentially expressed genes were used to draw a volcano map, construct a PPI protein interaction network, and perform gene ontology (GO), Kyoto Encyclopedia of Genes and Genomes (KEGG), gene set enrichment analysis (GSEA) and immune infiltration analysis to predict the function of KNL1 during UCEC progression. The prognostic data of TCGA and 108 clinical patients were used to analyze the correlation of KNL1 expression with the survival of patients, and KM survival curves were drawn. The UCEC cell lines Ishikawa and Hec-1-A were used to verify the function of KNL1.

**Results:**

KNL1 is significantly overexpressed in UCEC and is associated with a poor prognosis. KNL1 overexpression is closely related to cell mitosis, the cell cycle and other functions and is correlated with the International Federation of Gynecology and Obstetrics (FIGO) stage, histological grade and other characteristics of UCEC patients. Knockdown of KNL1 expression in UCEC cell lines can inhibit their proliferation, invasion, metastasis and other phenotypes.

**Conclusion:**

KNL1 is a prognostic and diagnostic biomarker associated with immune evasion in patients with UCEC.

## Introduction

Uterine corpus endometrial carcinoma (UCEC) has the second highest incidence among types of gynecologic cancer (1, 2). In contrast to other malignant tumors, endometrial cancer’s incidence and associated mortality have been increasing, and its age of onset has also demonstrated a pattern of becoming increasingly younger (3–5). Although the vast majority of patients with endometrial cancer are diagnosed at an early stage and have a good 5-year relative survival rate (1), patients with advanced or recurrent endometrial cancer have a poor response to therapy and a poor prognosis (6, 7). There is currently no screening test for UCEC, and its diagnosis is entirely based on symptoms; however, this approach has low specificity (8, 9). All of these elements highlight the lack of advances in the management of UCEC. The discovery and characterization of novel biomarkers for screening, diagnosing, and predicting the outcome of UCEC are crucial for patients with the disease.

Kinetochore Scaffold 1 (KNL1), also known as CASC5, D40, and AF15Q14, is primarily expressed in healthy testicles, various human cancer cell lines, and primary malignancies (10). It is a newly discovered member of the cancer testicular gene family and is located on chromosome 15 (11, 12). KNL1 can ensure high-fidelity chromosome segregation and is essential for maintaining mitosis (13–15). KNL1 has previously been identified as a possible lung adenocarcinoma driver gene (16). Experiments have shown that KNL1 can inhibit the apoptosis of colorectal cancer cells and promote their proliferation (17). Meanwhile, knockdown of KNL1 expression in cervical cancer HeLa cells inhibited their proliferation and induced apoptosis both *in vivo* and *in vitro (* 18). All of the aforementioned findings imply that KNL1 may be crucial to the emergence, growth, and progression of various malignancies.

Nevertheless, there has not been enough research to conclusively show that KNL1 is involved in the emergence and progression of UCEC. In this study, The Cancer Genome Atlas (TCGA) and the Gene Expression Omnibus (GEO) databases were used to analyze the expression of KNL1 and its correlation with clinical features, and the immunohistochemical results of 108 clinical specimens of UCEC were used to verify its expression. At the same time, a protein−protein interaction (PPI) network was constructed using KNL1 and its related differentially expressed genes, and gene ontology (GO), Kyoto Encyclopedia of Genes and Genomes (KEGG), gene set enrichment analysis (GSEA) and immune infiltration analysis were performed to predict the function of KNL1 in promoting the occurrence and development of UCEC. Finally, the UCEC cell lines Ishikawa and Hec-1-A were used to verify the function of KNL1 and clarify the molecular mechanism by which KNL1 promotes the progression of UCEC.

## Methods and materials

### Data sources and preprocessing

RNA-seq data from the TCGA (https://portal.gdc.cancer.gov/) UCEC project and GTEx database describe the differential expression of KNL1 in unpaired and paired samples. The Toil process uniformized the data (19). The TCGA level 3 HTSeq-FPKM (Fragments Per Kilobase Per Million) format was translated to the TPM (transcripts per million reads) format and log2-transformed. All final TCGA-based analyses were conducted using TPM-formatted data. Using GEOquery [version 2.54.1] (20), the differential analysis data for KNL1 in dataset GSE17025 (21, 22) were extracted from the GEO database. These data were obtained by removing probes corresponding to multiple molecules, and when probes corresponding to the same molecule were encountered, only the probe with the highest signal value was retained. The data were then normalized once more using the normalize Between Arrays function of the limma package [version 3.42.2] (23). Using the CPTAC database in UALCAN (http://ualcan.path.uab.edu) (24, 25), differential expression of the KNL1 protein in UCEC and normal adjacent tissues was determined. R was used for all statistical analyses and visualizations (version 3.6.3).

### Single-gene differential analysis and correlation analysis of KNL1

The DESeq2 package [version 1.26.0] and the STAT package [version 3.6.3] were used to conduct single-gene differential analysis and single-gene correlation analysis of KNL1 in the UCEC project utilizing the TCGA database (26). The findings of the single-gene differential analysis were used to generate volcano plots with the ggplot2 software [version 3.3.3]. |log_2_ fold change (LogFC)|>1 and p.adj<0.05 were used as the thresholds for differentially expressed genes (DEGs). The STRING database was utilized to show the DEGs (27), the PPI network of DEGs was analyzed using the Cytoscape program, and the MCODE plugin was used to identify the HUB genes. The genes from the single-gene correlation analysis were then sorted by |Pearson value| in descending order, and the top 50 correlations were retrieved. The KNL1 single-gene coexpression heatmap was generated using the top 50 genes and the HUB gene by the ggplot2 [version 3.3.3] package.

### Functional enrichment analysis

In the TCGA UCEC project, gene set enrichment analysis (GSEA) was utilized to investigate the putative signaling pathways based on differential expression analysis (KNL1 high-expression vs. KNL1 low-expression samples). The reference gene set was h.all.v7.2.symbols.gmt [Hallmarks]. An adjusted p value <0.05 was considered significantly enriched. After screening the DEGs based on the threshold (|LogFC |>1 and p.adj<0.05), Gene Ontology (GO) and Kyoto Encyclopedia of Genes and Genomes (KEGG) analyses were performed to enhance the pathways associated with KNL1 in UCEC using the R packages “clusterProfiler” and “org.Hs.eg.db”. p.adj<0.05 was considered significantly enriched.

### Immunoinfiltration analysis of KNL1

GSVA [version 1.34.0] was used to examine the relative infiltration levels of 24 immune cells (28). For the immune infiltration algorithm, ssGSEA was employed, and Spearman correlation analysis was applied. The markers for twenty-four immune cells were derived from an article in Immunity (29). The samples were then separated into low and high KNL1 expression groups, the enrichment scores of various immune cell infiltrates in the various subgroups were computed, and the analysis was conducted using GSVA software [version 1.34.0]. Finally, the correlation between KNL1 and CD47, CD273, and TNFRSF4 was computed, and ggplot2 software [version 3.3.3] was used to depict it.

### Analysis of the correlation between KNL1 mRNA expression and the prognosis of patients with UCEC

The survival data of UCEC patients were statistically analyzed using the survival package [version 3.2-10], and the results were visualized using the survminer package [version 0.4.9] to plot the overall survival (OS), disease-specific survival (DSS), and progression-free interval (PFI) on Kaplan−Meier curves for the UCEC patients. Using the pROC package [version 1.17.0.1], ROC analysis was performed on the data to assess the accuracy of KNL1 for prognostication. All predictive data for the aforementioned survival study were from a Cell article (30). Finally, a dichotomous logistic regression model and clinical baseline datasheet were developed to predict the association between various clinicopathological characteristics and KNL1 expression.

### Specimens

Jilin University’s School of Nursing’s Ethical Review Committee authorized the present study (Changchun, China). Paraffin−embedded specimens were collected at the Second Hospital of Jilin University (Changchun, China) from 108 patients with UCEC and 15 normal controls diagnosed between December 2012 and December 2019. Patients were informed about the UCEC-related study and agreed to participate. The criteria for inclusion were: i) initially diagnosed with UCEC and treated with standard surgery and/or radiotherapy and/or chemotherapy according to the FIGO stage and pathological type of the individual patient; ii) the diagnosis of UCEC was determined by an experienced gynecological pathologist; iii) the postoperative pathology results were interpreted by an experienced gynecological pathologist using FIGO staging criteria (Version 2009); and iv) complete follow-up data were available. The exclusion criteria were: i) a personal history of other malignant tumors; ii) preoperative radiation, chemotherapy, or hormonotherapy; and iii) a secondary uterine tumor. As stated in **Supplementary Table 4**, accessible clinical/pathological data were gathered from The Second Hospital of Jilin University’s Medical Record Database. All 108 patients with UCEC were followed up, and their OS was determined.

### Cell culture and stably transfected cell line development

The human UCEC cell lines Ishikawa and HEC-1-A were purchased from iCell Bioscience Inc., Shanghai. Ishikawa cells were cultured with Minimum Essential Medium (MEM, product code iCell-0012, iCell) supplemented with 10% fetal bovine serum (product code FS301-02; TransGen), 1% nonessential amino acids (NEAA, product code iCell-01000, iCell) and 1% penicillin‐streptomycin (product code P1400, Solarbio). HEC-1-A cells were cultured with McCoy’s 5A medium (product code iCell-0011, iCell) supplemented with 10% fetal bovine serum and 1% penicillin‐streptomycin. The two cell lines were cultured at 37°C in a humidified atmosphere with 5% CO_2_.

The lentiviral vector plasmid pLKO.1-Puro (product code FH1717; Hunan Fenghui Biotechnology Co., Ltd.) was utilized to construct the pLKO.1-Scramble and pLKO.1-shKNL1 plasmids. The interference sequences were 5’-GGUAAAAGUCCCAUAGAAATT-3’ for shKNL1 and 5’-GTATAAGTCAACTGTTGAC-3’ for shScramble. The lentiviruses used in this study were packaged using the 3 plasmid packaging system. After combining the lentiviral vector plasmids with the packaging plasmid PMD2.G (product code BR037, Fenghui), psPAX2 (product code BR036, Fenghui) and Lipofectamine™ 3000 transfection reagent (product code L3000150, Thermo Fisher), the complexed solution was introduced to HEK-293T cells (product code iCell-h237, iCell). The medium was collected and filtered using a 0.22 μm filter after 48 and 72 hours. The medium was then kept at 4°C for up to one week before use.

To generate stably transfected cell lines, Ishikawa and HEC-1-A cells were seeded into 6-well plates (300,000 cells/well). Subsequently, 24 hours later, 1 ml of medium containing the above lentivirus was added to each well. After 48 hours, the medium was changed. Cells infected with viruses encoding the puromycin resistance gene were selected in 2 μg/mL puromycin. One week of puromycin selection was continued prior to cell collection and subsequent analysis.

### Immunohistochemistry

Similar to an earlier study (31), immunohistochemical (IHC) staining was conducted. After 24 hours of fixation in 10% formalin at room temperature, the samples were embedded in paraffin and sectioned to a thickness of 3 µm. The sections were immersed in EDTA retrieval buffer (catalog number AR0023; Wuhan Boster Biological Technology, Ltd.) and cooked in a microwave. Then, 5% bovine serum albumin (product code AR1006; Boster Biological Technology, Inc.) was applied at room temperature for 20 minutes to prevent nonspecific binding. The histological sections were stained overnight at 4°C with rabbit anti-KNL1 antibody (product code DF13491; 1:100; Affinity), rabbit anti-CD56 antibody (product code GB112671; 1:750; Servicebio), rabbit anti-CD4 antibody (product code GB11064; 1:1000; Servicebio), and rabbit anti-B3GAT1 antibody (product code GB113461; 1:1000; Servicebio). The secondary antibody was goat antirabbit IgG coupled with horseradish peroxidase (product code GB23204; 1:200; Servicebio), and the staining technique was performed at 37°C for 30 minutes. Reactive products were observed using 3,3’diaminobenzidene (Boster Biological Technology, Inc.) as the chromogen, and the sections were counterstained for 2 minutes at room temperature with 0.1% hematoxylin (Boster Biological Technology, Inc.). Under a light microscope (AE2000, Motic) with an objective magnification of x200 or x400, images of the stained sections were recorded. The positive cell density was evaluated with Image-Pro Plus 6.0 (Media Cybernetics, Inc.), and the findings are reported as the average optical density (AOD) values. Two experienced pathologists from the Pathology Department of the Second Hospital of Jilin University graded the IHC staining independently under double-blind conditions.

### Real time-PCR

Total RNA was isolated from fresh frozen tissue and stably transfected cells using an EasyPure RNA kit (product code ER101-01; TransGen), and first-strand cDNA was synthesized using a cDNA synthesis kit (product code AT311-02; TransGen) following the manufacturer’s instructions. KNL1 transcription levels were determined by real-time PCR using the SYBR Green qPCR kit (product code AQ132; TransGen) according to the manufacturer’s instructions, with GAPDH serving as the internal reference gene. The following primers were used: KNL1 gene, 5’-GATGGGGTGTCTTCAGAGGC-3’ for forward and 5’-AGAGGACTCCTTGGGGGTTT-3’ for reverse; GAPDH gene, 5’‐GAAGGTGAAGGTCGGAGTC‐3’ for forward and 5’‐GAAGATGGTGATGGGATTTC‐3’ for reverse. An ABI-Q3 was used to conduct PCR at 94°C for 30 seconds, followed by 45 cycles of amplification at 94°C for 5 seconds, 51°C for 15 seconds, and 72°C for 10 seconds (Thermo Fisher Scientific, Inc.). The expression levels of the mRNA were measured using the 2^-ΔΔCt^ method (31).

### Cell counting kit-8 assay

Ishikawa, HEC-1-A, Ishikawa-shScramble, HEC-1-A-shScramble, Ishikawa-shKNL1 and HEC-1-A-shKNL1 cells were seeded into 96-well plates (3,000 cells/well). CCK-8 reagent (10 μl/well; product number BA00208, Bioss) was then added to each well 24, 48, and 96 hours later. After 1.5 hours of culture at 37°C, the absorbance of each well was measured at 450 nm with a microplate reader (E0226; Detie, Inc.

### Invasion assay

For the invasion experiment, a Transwell chamber (Labselect, product code 14342) was used to determine the invasive potential of the UCEC cells listed above. The chamber was covered with Matrigel (BD Biosciences, product code 356234) per the manufacturer’s instructions. A total of 3x10^4^ cells in 100 μL serum-free MEM or McCoy’s 5A were placed in the upper chamber, while 600 μL 10% FBS media-based medium was placed in the lower chamber. After 30 hours of treatment at 37°C, the residual cells on the top surface were removed with a cotton swab, and the invasive cells were stained with 10% Giemsa. An optical microscope was used to record the images (AE2000, Motic).

### Wound-healing assay

A wound-healing experiment was performed to assess the migratory capacity of the UCEC cells described above. Cells seeded in six-well plates (3x10^5^ cells/well) were scratched with a 200 μl pipette tip to create a linear wound. The dislodged cells were washed and removed with PBS. Photographs were obtained using a digital camera and an optical microscope (Motic Corporation) to observe the movement of cells into the wounded region at 24 and 48 hours. All micrographs were obtained at the same magnification at the same time for each cell type.

### Colony formation assay

HEC-1-A and Ishikawa cells were seeded onto 6-well plates at a density of 100 cells/well. Ten days later, the formation of typical colonies was observed. The cells were fixed with methanol and stained with 10% Giemsa (Biotopped, China). The number of visible colonies was counted to evaluate the colony formation ability of the cells. All experiments were conducted in three replicates.

### Statistical analysis

The statistical analyses were conducted with the mean of three independent tests plus the standard deviation (SD). Statistical analyses were conducted utilizing SPSS 23.0 or R version 3.6.3; differences between groups were examined using one-factor analysis of variance (ANOVA) followed by Dunnett’s *post hoc* test, Kruskal−Wallis test, or Student’s t test. When p<0.05, differences were judged statistically significant. The Spearman correlation coefficient was calculated to determine the correlation between KNL1 and CD4, CD56 and B3GAT1.

## Results

### Differential expression of KNL1 in pancancer and UCEC

As shown in **Supplementary Figure 1A**, we found that in unpaired samples, the expression of KNL1 was higher in the following tumors than in normal tissues: adrenocortical carcinoma (ACC), bladder urothelial carcinoma (BLCA), breast invasive carcinoma (BRCA), cervical squamous cell carcinoma and endocervical adenocarcinoma (CESC), colon adenocarcinoma (COAD), lymphoid neoplasm diffuse large B-cell lymphoma (DLBC), esophageal carcinoma (ESCA), glioblastoma multiforme (GBM), head and neck squamous cell carcinoma (HNSC), brain lower grade glioma (LGG), liver hepatocellular carcinoma (LIHC), lung adenocarcinoma (LUAD), lung squamous cell carcinoma (LUSC), ovarian serous cystadenocarcinoma (OV), pancreatic adenocarcinoma (PAAD), prostate adenocarcinoma (PRAD), rectum adenocarcinoma (READ), skin cutaneous melanoma (SKCM), stomach adenocarcinoma (STAD), thyroid carcinoma (THCA), thymoma (THYM), uterine corpus endometrial carcinoma (UCEC), and uterine carcinosarcoma (UCS). Similarly, the expression of KNL1 was decreased in kidney renal clear cell carcinoma (KIRC), kidney renal papillary cell carcinoma (KIRP), acute myeloid leukemia (LAML), and testicular germ cell tumors (TGCTs) compared to normal tissues.

**Figure 1:**
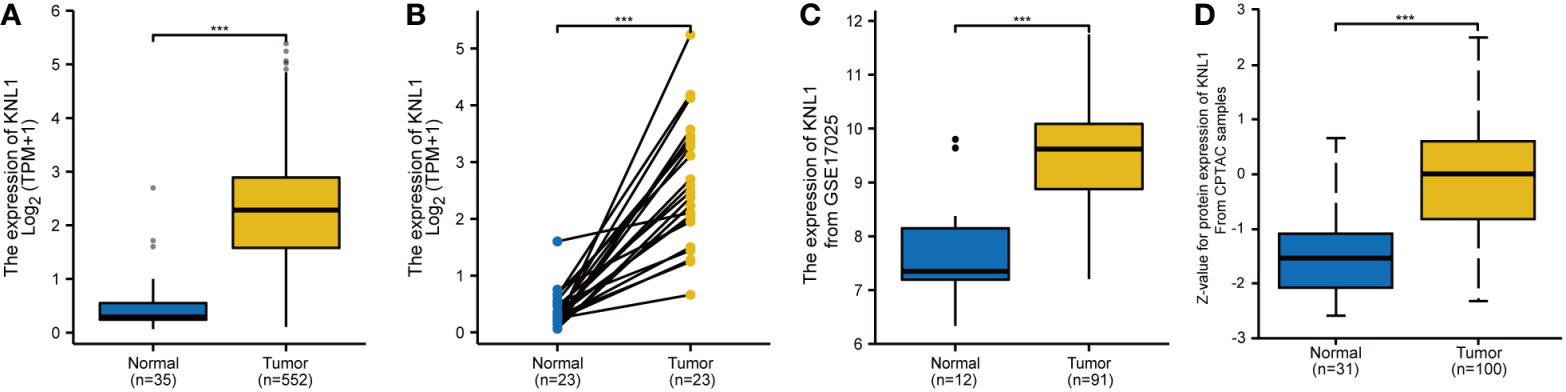
Differential expression analysis of KNL1 in patients with UCEC. **(A)** Differential analysis of KNL1 expression in unpaired UCEC samples. **(B)** Differential analysis of KNL1 expression in paired UCEC samples. **(C)** Differential analysis of KNL1 expression based on GSE17025 data. **(D)** Differential analysis of KNL1 protein expression based on CPTAC data. Significance identifier: ***, p<0.001.

As shown in **Supplementary Figure 1B**, in paired samples, the expression of KNL1 in BLCA, BRCA, COAD, ESCA, HNSC, LIHC, LUAD, LUSC, PRAD, STAD and UCEC was higher than that in adjacent tissues. The expression of KNL1 in KIRC and KIRP was lower than that in adjacent tissues. The number of tumor samples used in the pancancer analysis is presented in **Supplementary Table 1**. As shown in **Figures 1A, B**, in both unpaired and paired UCEC samples, the expression of KNL1 in tumors was higher than that in normal tissues. This point was validated by utilizing the mRNA and protein expression data of KNL1 in the GSE17025 and UALCAN databases, which were compatible with the results from the TCGA database, as shown in **Figures 1C, D**.

### Evaluation of the expression of KNL1 in clinical samples of UCEC

**Figure 2:**
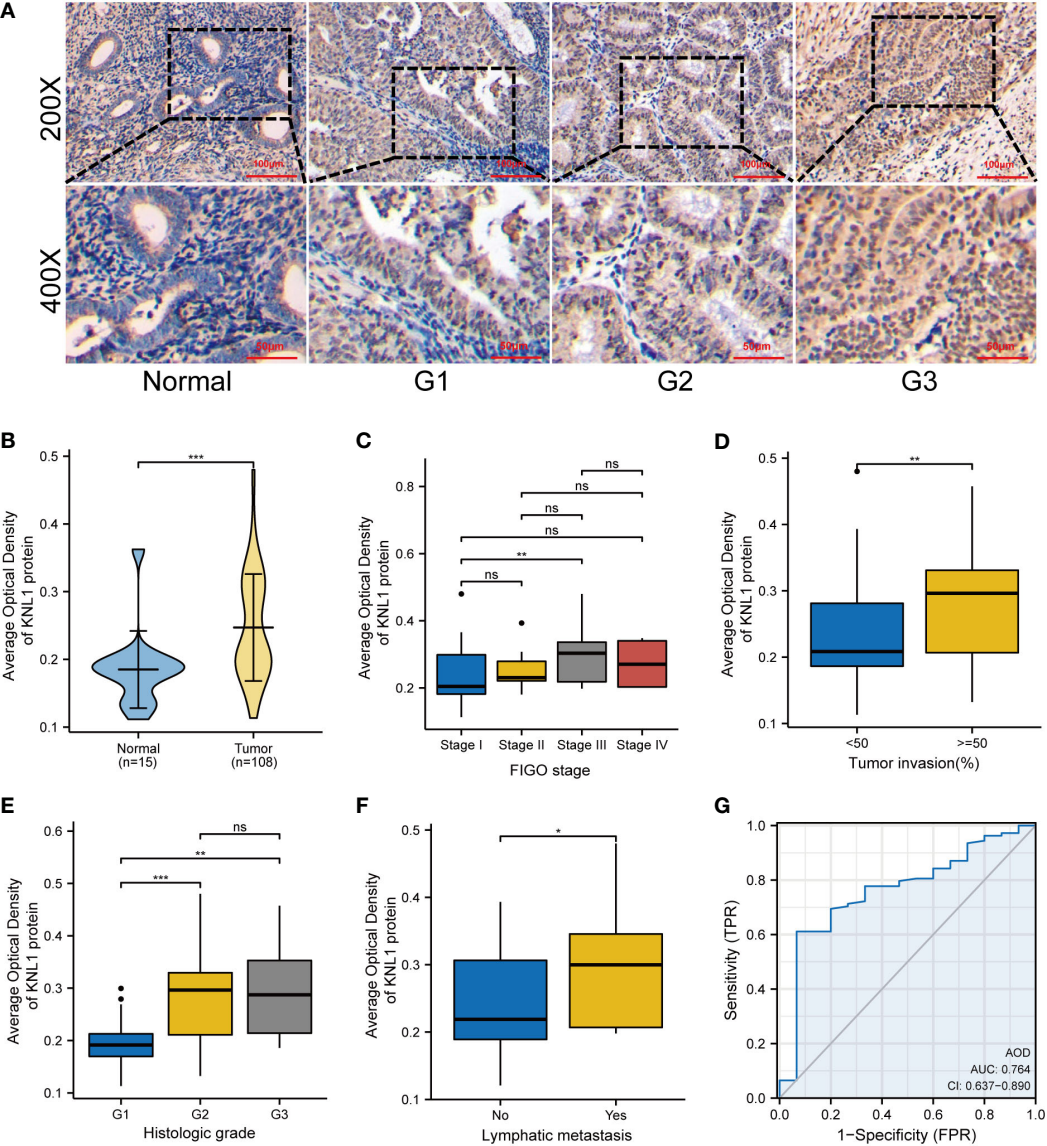
Expression and clinical correlation analysis of KNL1 in UCEC clinical samples. **(A)** Immunohistochemical results of KNL1 in normal endometrial tissues and UCEC tissues with different degrees of differentiation. **(B)** Group comparison of KNL1 immunohistochemical results in 108 UCEC clinical specimens and 15 normal endometrial cancer tissues. **(C–F)** Group comparison of KNL1 protein expression levels in samples with different clinical characteristics, **(C)** FIGO stage, **(D)** Tumor invasion, **(E)** Histologic grade, and **(F)** Lymphatic metastasis. **(G)** The diagnostic ROC curve of KNL1. Significance identifier: ns (no significance), p≥0.05; *, p< 0.05; **, p<0.01; ***, p<0.001.

The AOD of 108 UCEC clinical samples and 15 normal tissues was measured by immunohistochemical staining. As shown in **Figure 2A**, KNL1 expression was different in tissues with different degrees of differentiation. The expression of KNL1 protein in normal tissues is low, and the expression of KNL1 in tumor tissues gradually increases with a gradual decrease in tumor differentiation. The expression levels of KNL1 protein in 108 UCEC samples and 15 normal samples are shown in **Figure 2B**. The expression of KNL1 was significantly increased in tumor tissues. As shown in **Figures 2C–F**, KNL1 expression was different in patients with different FIGO stages, different tumor invasion statuses, different histologic grades, and different lymphatic metastases. A KNL1 expression box diagram of patients with other clinical features is shown in **Supplementary Figure 2**. Moreover, ROC curves of the protein expression data of KNL1 are shown in **Figure 2G**. The AUC=0.764, suggesting that KNL1 may be closely related to the occurrence and development of tumors.

### Single-gene differential analysis and correlation analysis of KNL1

**Figure 3:**
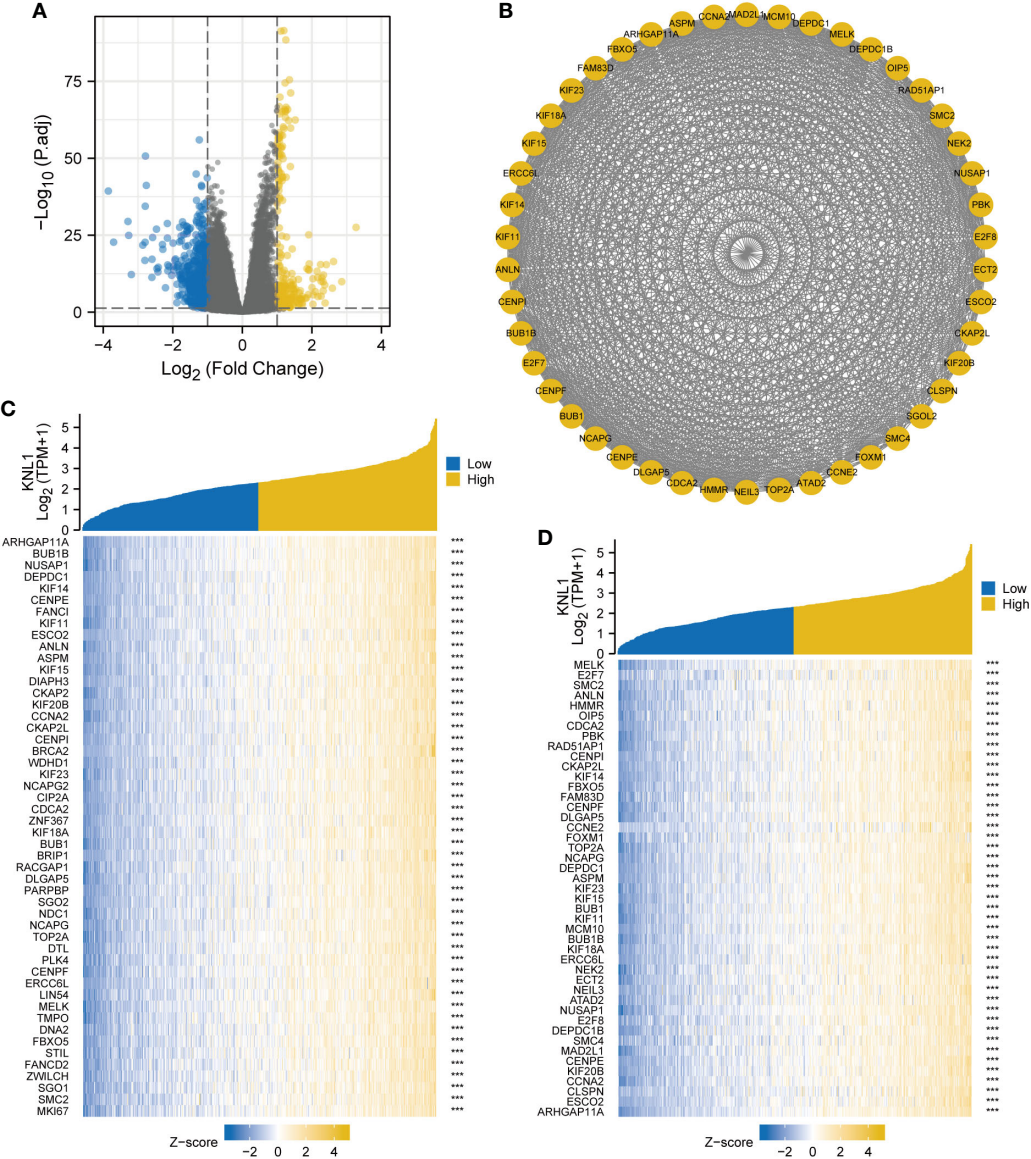
Single gene differential analysis and correlation analysis of KNL1. **(A)** Volcano map for single gene differential analysis of KNL1. **(B)** Protein interaction network diagram (PPI) of the HUB genes. **(C)** Heatmap of coexpression of the top 50 most correlated genes with KNL1 in single gene correlation analysis. **(D)** Heatmap of single gene coexpression of the HUB genes and KNL1.

The results of single-gene differential analysis are shown in the volcano plot in **Figure 3A**. There were 850 genes that satisfied the threshold of |LogFC|>1 and p.adj<0.05, under which 243 genes were highly expressed and 607 genes were poorly expressed. These 850 genes were imported into the STRING database to construct differential protein interaction networks, and a total of 46 HUB genes were identified (MELK, E2F7, SMC2, ANLN, HMMR, OIP5, CDCA2, PBK, RAD51AP1, CENPI, CKAP2L, KIF14, FBXO5, FAM83D, CENPF, DLGAP5, CCNE2, FOXM1, TOP2A, NCAPG, SGOL2, DEPDC1, ASPM, KIF23, KIF15, BUB1, KIF11, MCM10, BUB1B, KIF18A, ERCC6L, NEK2, ECT2, NEIL3, ATAD2, NUSAP1, E2F8, DEPDC1 B, SMC4, MAD2L1, CENPE, KIF20B, CCNA2, CLSPN, ESCO2, and ARHGAP11A), as shown in **Figure 3B**. After that, we performed a correlation analysis and created a coexpression heatmap utilizing the 50 genes with the strongest connection with KNL1, as shown in **Figure 3C**. The heatmap of coexpression between the HUB genes and KNL1 is shown in **Figure 3D**.

### Functional enrichment analysis of KNL1 in UCEC

**Figure 4:**
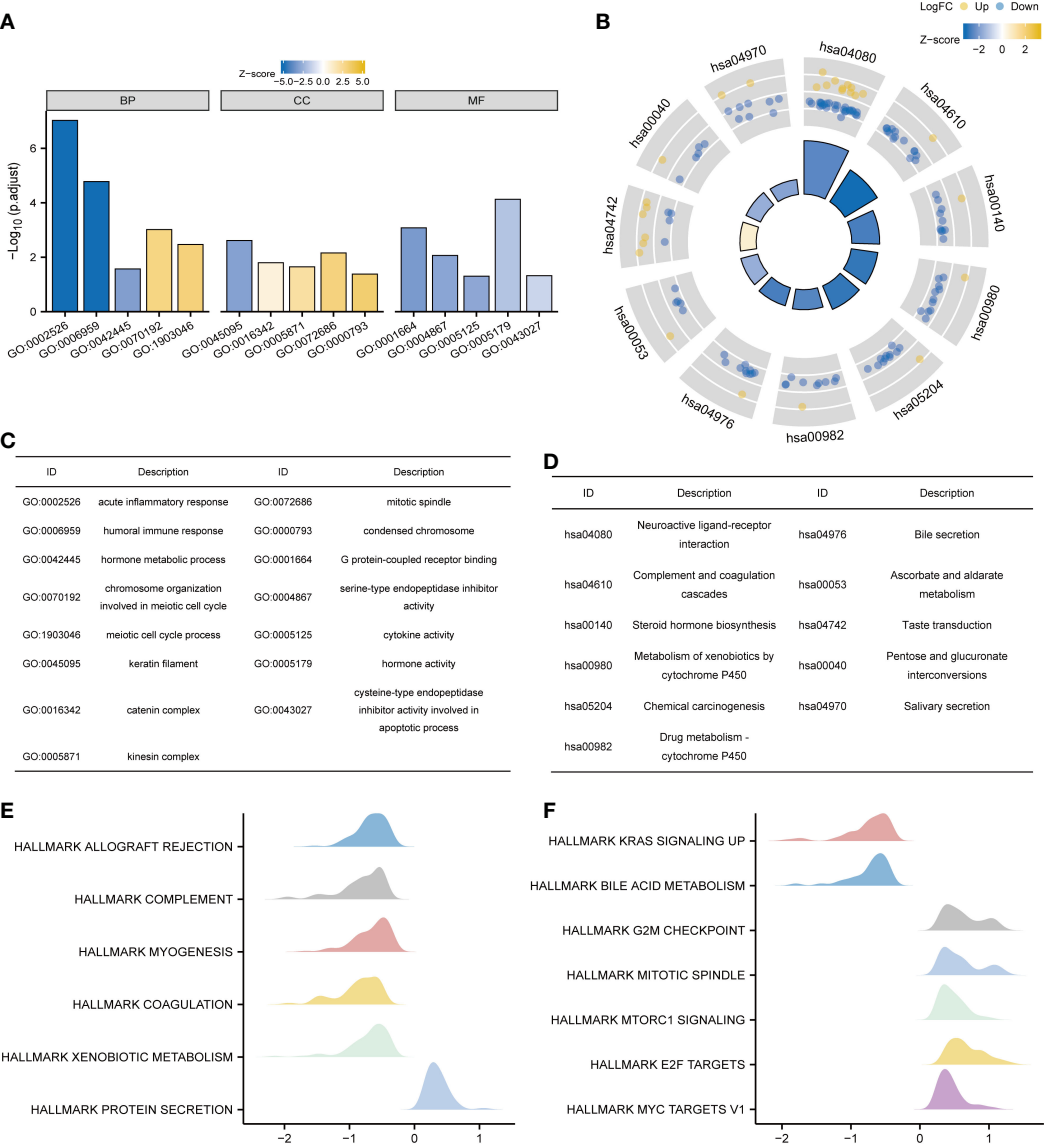
Functional enrichment analysis of KNL1 and related differentially expressed genes in UCEC. **(A)** Results of GO analysis. **(B)** Results of KEGG analysis. **(C, D)** GO and KEGG analysis category names corresponding to the GO and KEGG Identifier. **(E, F)** Results of KEGG analysis. When the abscissa was positive, KNL1 expression was positively correlated with this pathway, and when the abscissa was negative, the opposite was observed.

KNL1 and its differentially expressed genes were used for GO and KEGG functional enrichment analyses. GO functional enrichment analysis showed that in terms of “biological process”, pathways such as acute inflammatory response, humoral immune response, hormone metabolic process, chromosome organization involved in meiotic cell cycle, and meiotic cell cycle process were enriched. In terms of “molecular function”, significant enrichment occurred in the pathways of G protein-coupled receptor binding, serine-type endopeptidase inhibitor activity, cytokine activity, hormone activity, and cysteine-type endopeptidase inhibitor activity involved in the apoptotic process. In terms of “cellular component”, keratin filament, catenin complex, kinesin complex, mitotic spindle, condensed chromosome and other pathways were enriched, and the results are shown in **Figures 4A, C** and **Supplementary Table 2**.

The results of KEGG functional enrichment analysis showed that steroid hormone biosynthesis, metabolism of xenobiotics by cytochrome P450, drug metabolism - cytochrome P450, pentose and glucuronate interconversions, etc., were enriched, as shown in **Figures 4B, D** and **Supplementary Table 2**.

Finally, GSEA functional enrichment analysis was used to predict the function of KNL1 in the development of endometrial carcinoma, and it was found that KNL1 was closely associated with hallmark allograft rejection, hallmark complement, hallmark Kras signaling up, hallmark g2m checkpoint, hallmark mitotic spindle, hallmark mtorc1 signaling, hallmark e2f targets, hallmark myc targets v1 and other pathways, as shown in **Figures 4E, F**.

### Immunoinfiltration analysis of KNL1 in UCEC

**Figure 5:**
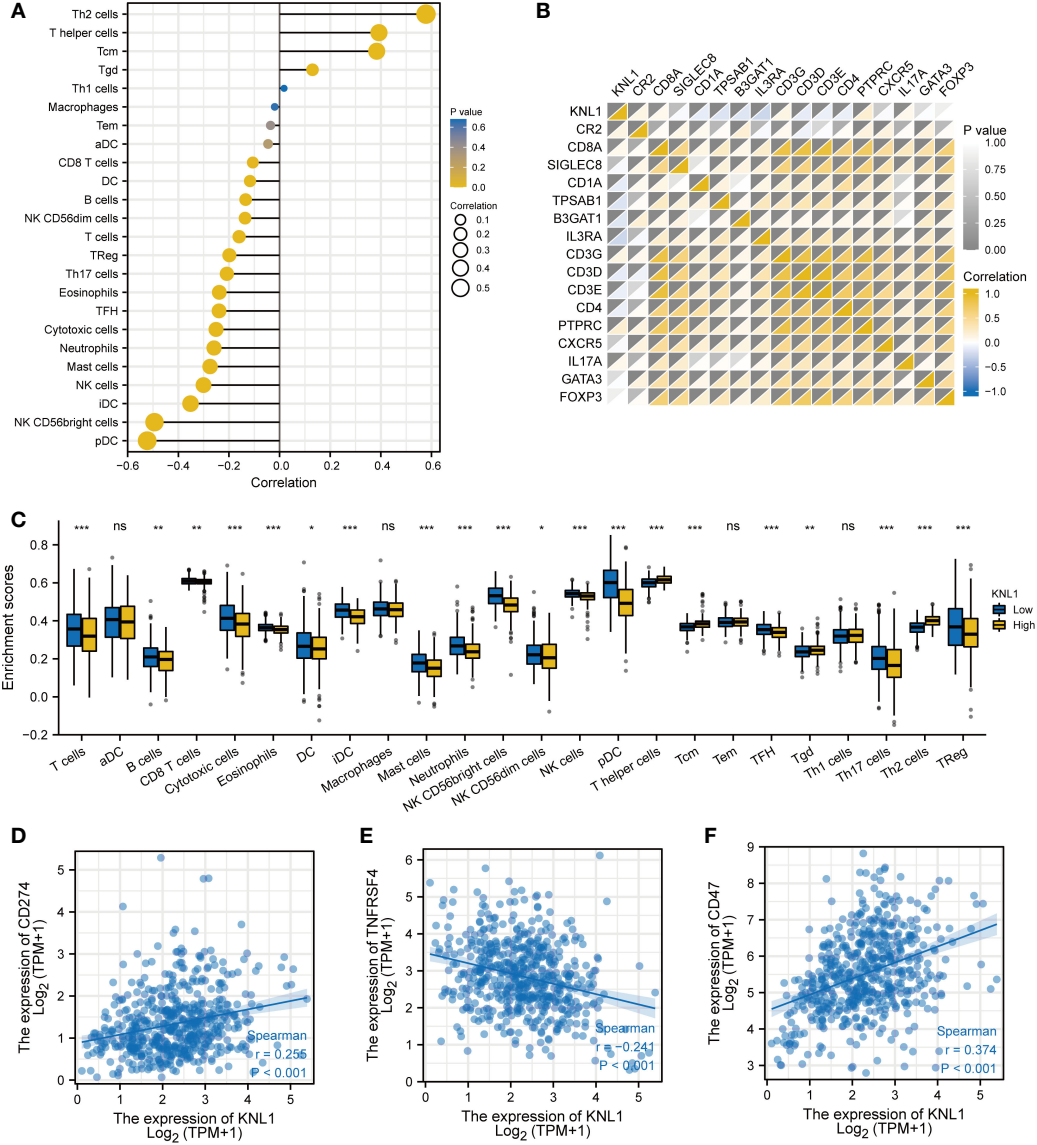
Immunoinfiltration analysis of KNL1. **(A)** Correlation analysis between KNL1 and 24 immune cell infiltration levels. **(B)** Heatmap of the correlation between KNL1 expression and various immune cell surface marker proteins: CR2 (B cells), CD8A (cytotoxic cells), SIGLEC8 (eosinophils), CD1A (iDCs), TPSAB1 (mast cells), B3GAT1 (NK cells), IL3RA (pDCs), CD3G (T cells), CD3D (T cells), CD3E (T cells), CD4 (T helper cells), PTPRC (Tcm), CXCR5 (Tfh), IL17A (Th17), GATA3 (Th2), and FOXP3 (Treg). **(C)** Infiltration levels of 24 kinds of immune cells in samples with different KNL1 expression levels. **(D–F)** Correlation analysis between KNL1 and the expression levels of CD274, TNFRSF4 and CD47. Significance identifier: ns (no significance), p≥0.05; *, p< 0.05; **, p<0.01; ***, p<0.001.

The relationship between the expression of KNL1 and the degree of infiltration of 24 immune cells was analyzed, and the results are shown in **Figures 5A, C**. The results showed that the expression of KNL1 had a significant positive correlation with the infiltration degree of Th2 cells, T helper cells, and Tcm cells, while the expression of KNL1 showed a significant negative correlation with the infiltration degree of pDCs, NK CD56bright cells, iDCs, and NK cells.

To validate the results of ssGSEA, we analyzed the correlation between the expression of KNL1 and the expression of various immune cell surface marker proteins and plotted a heatmap, shown in **Figure 5B**. The heatmap showed a strong correlation between KNL1 and CR2, CD1A, TPSAB1, B3GAT1, IL3RA, CD3D, and PTPRC, consistent with the previous analysis. Finally, as shown in **Figures 5D–F**, we analyzed the correlation between some common immunotherapeutic targets and KNL1 and found that the expression of KNL1 showed a significant positive correlation with the expression of CD47.

**Figure 6:**
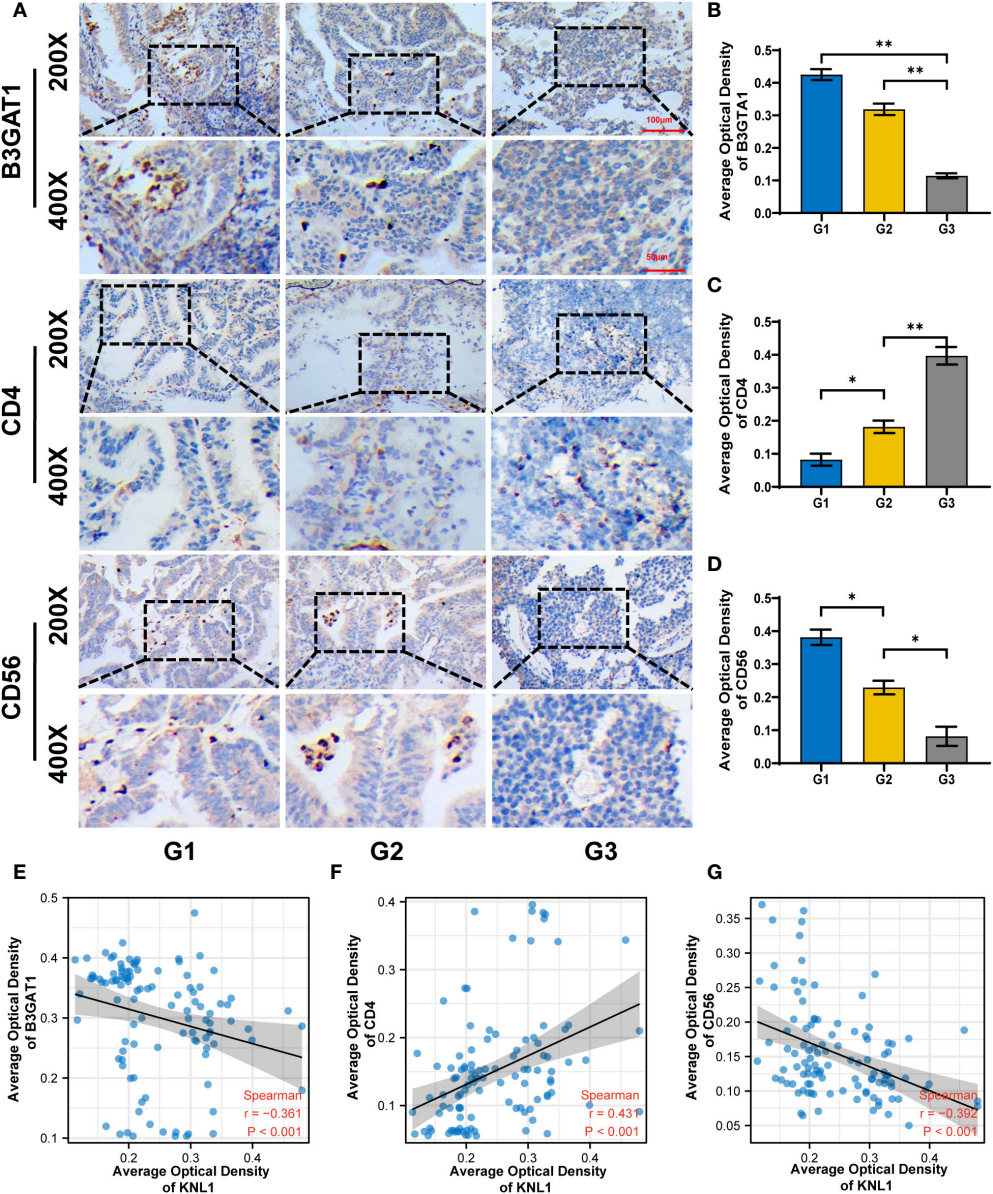
Correlation between KNL1 expression and the expression of CD4, CD56 and B3TAG1 in patients with UCEC. **(A)** Immunohistochemical images of CD4, CD56 and B4GAT1 in UCEC patients with different histologic grades. **(B–D)** Histogram of the immunohistochemical results for CD4, CD56, and B4GAT1. **(E–G)** Scatter plot of the correlation between the expression levels of CD4, CD56, and B3GAT1 and KNL1. Significance identifier:p≥0.05; *, p< 0.05; **, p<0.01.

To verify the relationship between KNL1 expression and immune infiltration in patients with UCEC, immunohistochemical analysis of CD4, CD56 and B3GAT1 was performed using samples from 108 patients, shown in **Figures 6A–D**. The immunohistochemical results were used to analyze the correlation between KNL1 and CD4, CD56 and B3GAT1, and it was found that the expression of KNL1 was negatively correlated with the expression of CD56 and B4GAT1 and positively correlated with the expression of CD4, as shown in **Figures 6E–G**.

### Effect of KNL1 expression on the prognosis of tumor patients

**Figure 7:**
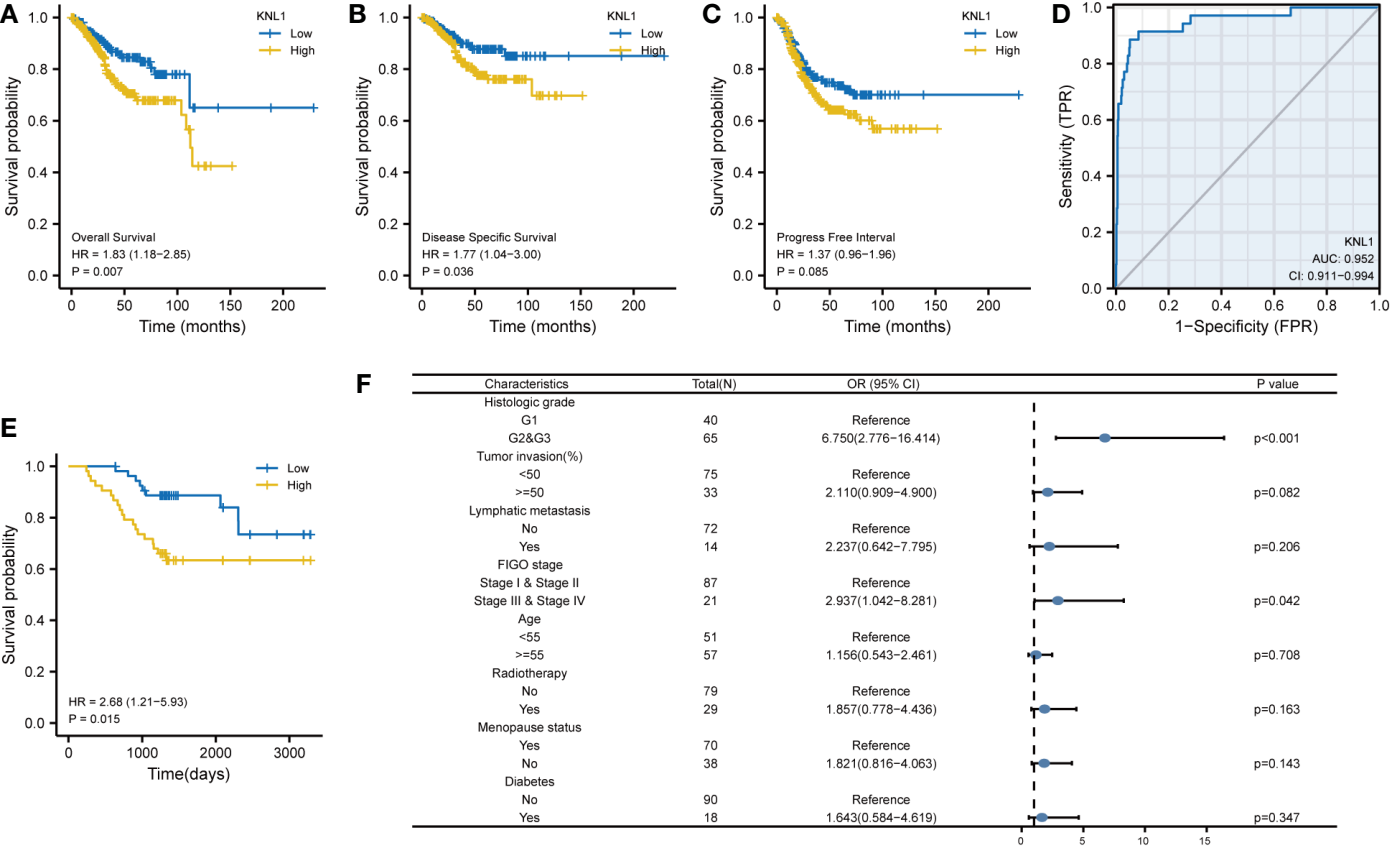
Correlation of KNL1 expression with the outcomes of UCEC patients. **(A–C)** KM survival curves stratified by KNL1 expression for overall survival (OS), disease-specific survival (DSS), and progression-free interval (PFI). **(D)** Prognostic ROC curve; the area under the ROC curve was between 0.5 and 1. The closer the AUC is to 1, the better the diagnostic effect is. The AUC has a low accuracy when it is between 0.5 and 0.7, a moderate accuracy when it is between 0.7 and 0.9, and a high accuracy when it is above 0.9. **(E)** KM OS curves stratified by KNL1 expression of 108 clinical samples of UCEC. **(F)** Results of binary logistic regression analysis of the correlation between the KNL1 expression level and the clinical characteristics of the 108 patients. The data were incomplete, as some records were lost.

To determine the relationship between KNL1 expression and the prognosis of UCEC patients, we performed survival analysis using the prognostic data of UCEC in TCGA, and the results are shown in **Figures 7A–C**. We found that high expression of KNL1 was correlated with worse overall survival (OS), disease-specific survival (DSS) and progression-free interval (PFI). As shown in **Figure 7D**, we also performed a ROC analysis to test the accuracy of KNL1 expression in predicting patient outcome and found that the AUC=0.952, suggesting that KNL1 expression is highly accurate in predicting the outcome of UCEC patients.

After that, we analyzed the relationship between KNL1 expression and various clinical characteristics of UCEC patients, for which the baseline data table is shown in **Supplementary Table 3**, and the results of the logistic analysis are shown in **Table 1**. The results in both **Supplementary Table 3** and **Table 1** suggest that the expression of KNL1 is closely related to the histologic grade of UCEC patients.

**Table 1:** The results of the logistic regression model obtained from the RNA-seq data in the TCGA database.

Characteristics	Total(N)^a^	Odds Ratio(OR)	P value
Clinical stage (Stage IV & Stage II & Stage III vs. Stage I)	552	1.361 (0.965-1.924)	0.080
Age (>60 vs. <=60)	549	1.038 (0.734-1.466)	0.834
BMI (>30 vs. <=30)	519	0.950 (0.669-1.348)	0.773
Histological type (Mixed & Serous vs. Endometrioid)	552	1.121 (0.765-1.644)	0.559
Histologic grade (G2 & G3 vs. G1)	541	3.395 (2.114-5.605)	<0.001
Tumor invasion(%) (>=50 vs. <50)	474	0.976 (0.679-1.403)	0.898
Menopause status (Post vs. Pre & Peri)	506	0.759 (0.423-1.349)	0.349
Diabetes (Yes vs. No)	451	1.206 (0.796-1.829)	0.377

^a^Data incomplete as some record data were lost.

Finally, in addition to analyzing the correlation between KNL1 expression and the prognosis of UCEC patients, we also used the prognostic data of GBMLGG, LGG, BRCA, KIRP, KIRC, and PAAD in the TCGA database to analyze the association of KNL1 expression with the prognosis of these tumors, and the results are shown in **Supplementary Figures 3A–F**. The results showed that high expression of KNL1 led to worse OS of patients with these tumors, suggesting that KNL1 may be closely related to tumor progression.

We then analyzed the relationship between KNL1 expression and clinical features using the clinical information of 108 previously collected samples to verify the relationship between KNL1 and the prognosis of UCEC patients, and the results are shown in **Figures 7E, F**. The results in Figure E show that high expression of KNL1 was correlated with a poor prognosis in these 108 clinical patients, which further verifies the relationship between KNL1 expression and the OS of patients. Figure F shows the results of logistic regression analysis, indicating that there is a relationship between KNL1 expression and FIGO stage and histologic grade. The results in **Supplementary Table 4** also show that the expression of KNL1 is significantly related to the degree of histologic grade, tumor invasion, FIGO stage and the expression of Ki67 protein, which is consistent with the previous analysis.

### Effect of KNL1 knockdown on the proliferation, invasion and metastasis of endometrial cancer cells

**Figure 8:**
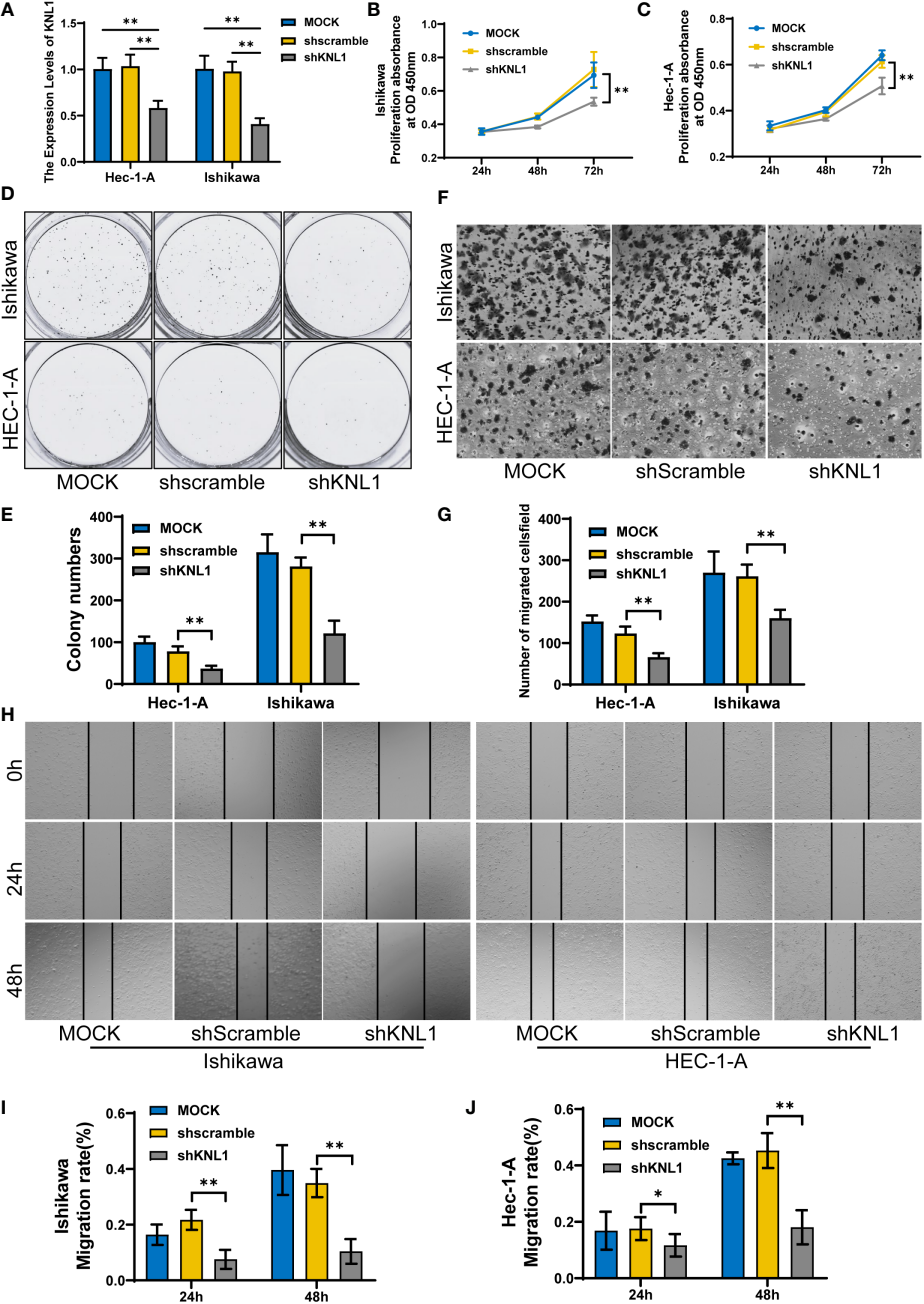
Effect of knockdown of KNL1 expression in UCEC cell lines on tumor cell proliferation, invasion and other phenotypes. **(A)** Ishikawa and Hec-1-A cells were transfected with shKNL1, and the level of KNL1 was evaluated by qRT−PCR. **(B, C)** The proliferation of Ishikawa and Hec-1-A cells was examined by CCK-8 **(D, E)** and colony-formation assays. **(F, G)** The migration of Ishikawa and Hec-1-A cells was examined by Transwell assays. **(H–J)** The metastatic capacity of Ishikawa and Hec-1-A cells was examined by wound-healing assays. Significance identifier: p≥0.05; *, p< 0.05; **, p<0.01.

After knocking down the expression of KNL1 in HEC-1-A and Ishikawa cell lines, the expression of KNL1 was confirmed to be significantly reduced, as shown in **Figure 8A**. We then performed CCK-8 assays and found that cell proliferation was significantly reduced after knockdown, as shown in **Figures 8B, C**. We also performed a wound-healing assay and found that the metastatic ability of cells with KNL1 knockdown was significantly weaker than that of the control group over time, as shown in **Figures 8H–J**. We also performed Transwell experiments and the invasive ability of cells with KNL1 knockdown was also significantly weakened, as shown in **Figures 8F, G**. Finally, we performed a colony formation assay and found that knockdown of KNL1 expression in cells was followed by a decrease in their colony formation ability **Figures 8D, E**).

## Discussion

To date, there is a lack of good biomarkers for screening and diagnosing UCEC (32). Finding and identifying new biomarkers for early screening and diagnosis is particularly important for patients at high risk of UCEC (33, 34). In addition, due to the limitations of clinical staging, the final pathological diagnosis and staging are based on surgical specimens (35, 36). Therefore, it is also necessary to study the prognostic biological indicators of UCEC, which will help to classify UCEC patients into low-risk and high-risk groups before surgery to improve individualized treatment (37).

In this study, using RNA-seq data from the TCGA and GEO databases, it was found that KNL1 was highly expressed in UCEC, suggesting that KNL1 was related to the occurrence and development of UCEC. Using 108 cases of endometrial carcinoma and 15 cases of normal endometrium, we found that the expression of KNL1 protein in tumors was higher than that in normal tissues. Its expression level was related to FIGO stage, tumor invasion, histologic grade and lymphatic metastasis. These results suggest that KNL1 may be a useful diagnostic molecular marker for UCEC and could predict the outcome of patients with UCEC. In addition, the ROC diagnostic curve drawn with the data obtained from the clinical samples showed that the AUC=0.764, further indicating that KNL1 could be useful in UCEC diagnosis.

To clarify the role of KNL1 in the occurrence and development of UCEC, 46 HUB genes closely related to the function of KNL1 and the most relevant 50 genes were identified by single gene differential analysis and single gene correlation analysis, including the KIF protein family, SMC protein family, BUB protein family, MELK and CENPF. Previous studies have found that CENPF, MELK, PBK, TOP2A and NEK2 are upregulated in breast cancer and this is associated with a poor prognosis. CENPF, MELK and PBK are related to CD4+ T cells, and TOP2A is related to CD8+ T cells (38, 39). In addition, MELK regulates cell cycle progression (40), leading to a worse prognosis in patients with adrenal cortical carcinoma and Wilms tumor (41, 42), and it could be a novel target for cancer therapy (43). The expression of E2F family proteins and BUB family proteins is also significantly related to the cell cycle and can promote the proliferation of tumor cells (44–47). E2F family proteins have also been shown to be potential targets for molecular diagnosis and targeted therapy of clear cell carcinoma and liver cancer (48). The expression of the SMC family is closely associated with B cells, CD4+ T cells, CD8+ T cells, macrophages, neutrophils, and DCs (49), which can be potential therapeutic targets for HCC, and it has been demonstrated that inhibitors targeting SMC2, SMC3, and SMC4 can be a practical therapeutic strategy for HCC (50, 51). All of the above results suggest that KNL1 may participate in cell mitosis and the cell cycle and thus play an important role in the occurrence and development of tumors.

To further understand the molecular mechanism of KNL1 in tumorigenesis and development, functional enrichment analysis of GO, KEGG and GSEA was performed using KNL1 and its related differentially expressed genes. GO analysis showed that KNL1 was involved in the humoral immune response, keratin filament, mitotic spindle and other biological processes. There is increasing evidence that the humoral immune response is associated with tumorigenesis (52). As a cytoskeletal protein of epithelial cells, keratin is involved in regulating apoptosis, growth and migration of tumor cells. An elevated level of keratin in the serum or tumor tissue of tumor patients has been used for the clinical diagnosis of tumors, and the expression level of keratin is negatively correlated with the survival of tumor patients and can be used as a prognostic marker (53–57).

The correct arrangement of mitotic spindles during cell division is essential for cell fate determination, tissue organization, and development. Changes in the dynamics and control of the microtubules that compromise the mitotic spindle leads to chromosomal instability, which in turn leads to the production of tumor cells (58, 59).

KEGG analysis also showed that KNL1 function was related to the biosynthesis of steroid hormones, the metabolism of cytochrome P450 and other pathways. Estrogen, as a steroid hormone, can bind to estrogen receptors and affect the progression of endometrial cancer (60). Previous studies have reported that high expression of cytochrome P450 can induce the development of tumors and inactivate anticancer drugs (61).

Consistent with the results of the GO analysis, the results of GSEA also showed that KNL1 was significantly enriched in many pathways related to mitosis. KNL1 is also closely related to the functions of the KRAS, mTORC1 and MYC genes. Previous studies have found that the KRAS gene acts as a switch in the body, regulating signaling pathways such as tumor cell growth and angiogenesis. Mutations in the KRAS gene cause continuous stimulation of cell growth, leading to tumorigenesis (62). mTORC1 can regulate cell proliferation, metabolism and survival by integrating growth factor signals and cell energy status. mTORC1 dysfunction plays a key role in tumor cell proliferation and metastasis (63). As a transcription factor with extensive functions, MYC is mainly activated by amplification, chromosomal translocation and rearrangement, regulates cell differentiation and proliferation through various mechanisms, and participates in the occurrence, development and evolution of tumors (64).

Given the correlation between KNL1-related genes and T cells, this study further explored the relationship between KNL1 and immune cell infiltration in tumors. KNL1 was positively correlated with the infiltration of Th2 cells, T helper cells and Tcm cells and negatively correlated with the infiltration of pDCs, iDCs and NK cells. This result was confirmed by immunohistochemical analysis of 108 endometrial carcinoma samples. Studies have shown that pDCs can promote the antitumor immune response (65), iDCs can promote the activation of T cells, and NK cells play a key role in immune regulation through interactions with DCs (66). During tumor progression, the transition from Th1/Th2 balance to Th2 dominance is crucial. Th2 cells are not conducive to cellular immune antitumor effects. Restoring the Th1/Th2 balance is of great significance in tumor therapy (67). The results of this study indicate that upregulation of KNL1 expression may be adverse to the antitumor immune response of the body, and it is significantly positively correlated with the immunotherapy target CD47, suggesting that KNL1 may be a potential immunotherapy target for tumor immunotherapy. Additional proteomics and larger sample size studies are needed for further verification of this possibility in the future.

Taking into consideration the upregulation of KNL1 expression in tumor tissues and its inhibition of antitumor immunity, we speculated that KNL1 might be correlated with the prognosis of patients with endometrial cancer. Using KNL1 expression network data and clinical data, KM survival analysis showed that high KNL1 expression predicted a poor prognosis. The ROC curve analysis showed that KNL1 had a high accuracy in predicting the outcomes of patients.

When analyzing the correlation between KNL1 expression and the clinical characteristics of patients, this study found that the expression of KNL1 was only correlated with the histologic grade of patients by using RNA-seq data analysis from online databases. However, immunohistochemical analysis of 108 clinical samples showed that KNL1 protein expression was correlated with FIGO stage, tumor invasion, histologic grades and lymphatic metastases of patients. The inconsistency between these results may be because the former was obtained from an analysis of RNA-seq data at the transcription level, while the latter was obtained from immunohistochemical analysis results at the protein level. The mRNA abundance does not necessarily have a linear relationship with the protein expression level of its translated products. There are many levels of regulation of protein content, and the transcription level is only one level. In addition, mRNA degradation, protein degradation, protein modification, protein folding and other factors may cause the mRNA abundance and protein expression levels to be inconsistent. These factors can all lead to differences in the final results (68). Meanwhile, the protein expression level in this study was quantified using the results of immunohistochemical analysis, and the sample size used in the analysis was only 108 cases, which may lead to bias in the analysis results. More proteomics and larger sample size studies are needed in the future to verify the relationship between the protein expression level of KNL1 and the clinical characteristics of patients.

Finally, to verify the function of KNL1, this study used the endometrial cancer cell lines HEC-1-A and Ishikawa to downregulate the expression of KNL1 by stable transfection of shRNA. Knockdown of KNL1 expression weakened cell viability and decreased the metastatic and invasive abilities of the tumor cells. This result further verified that KNL1 is closely related to the occurrence and development of tumors and is involved in the invasion and metastasis of tumor cells. Therefore, KNL1 can be used as a potential molecular target for tumor therapy.

In conclusion, the upregulation of KNL1 expression can promote the occurrence, metastasis and invasion of UCEC and inhibit the antitumor immune response. Therefore, KNL1 can be used as an independent risk factor for UCEC and is a potential molecular marker for diagnosing, treating and predicting the outcome of UCEC, which can help doctors make more reasonable treatment plans for patients.

At the same time, this study has certain limitations. First, there is a large difference between the numbers of tumor samples and normal samples, and further research is needed to narrow this difference in sample sizes in the future. In addition, the application of a single biomarker is unlikely to be sufficiently accurate for prognostication and diagnosis, and a combination of several different biomarkers needs to be further evaluated in the future. This can lead to the identification of algorithms with better diagnostic characteristics. This study verified the effect of KNL1 on UCEC cells, but the results related to pathway enrichment still need to be further verified by *in vitro* and *in vivo* experiments. This study is a retrospective study, and more prospective studies are needed in the future to reduce the bias inherently caused by retrospective studies.

## Data availability statement

Publicly available datasets were analyzed in this study. This data can be found here: The TCGA database (https://portal.gdc.cancer.gov/), The UALCAN database (http://ualcan.path.uab.edu), and GSE17025 of The GEO database (https://www.ncbi.nlm.nih.gov/geo/).

## Ethics statement

The present study was approved by the Ethics Committee of the School of Nursing, Jilin University (Changchun, China). The patients/participants provided their written informed consent to participate in this study. Written informed consent was obtained from the individual(s) for the publication of any potentially identifiable images or data included in this article.

## Author contributions

SZ and LZ conceived and designed the study. KH, JL acquired the data performed the statistical analysis. KW and XH performed the experiments and analysis the data. WZ and TW drafted the manuscript. JC, ZW and JY contributed to revising the manuscript for intellectual content and language editing. All authors contributed to the article and approved the submitted version.

## Supplementary material

Supplementary Figure 1 Differential expression analysis results of KNL1 in pancancer patients. **(A)** Results of differential analysis of KNL1 expression in 33 tumors based on TCGA database data. **(B)** Pancancer analysis of paired samples based on data from the TCGA database.

Supplementary Figure 2 Expression of KNL1 in UCEC patients with different histological types. **(A)** Box diagram of KNL1 expression obtained from RNA-seq data in the TCGA database. **(B)** Box plot of KNL1 expression using immunohistochemical analysis of 108 clinical samples.

Supplementary Figure 3 KM overall survival curves stratified by KNL1 expression in different tumors. **(A)** KM survival curve of GBMLGG, **(B)** KM survival curve of LGG, **(C)** KM survival curve of BRCA, **(D)** KM survival curve of KIRP, **(E)** KM survival curve of KIRC, and (F) KM survival curve of PAAD.
