# Supplementary figures and images for: KNL1 is a prognostic and diagnostic biomarker related to immune infiltration in patients with uterine corpus endometrial carcinoma
Source: Front Oncol. 2023 Jan 27;13:1090779. doi: 10.3389/fonc.2023.1090779 (PMC9913269; doi:10.3389/fonc.2023.1090779)

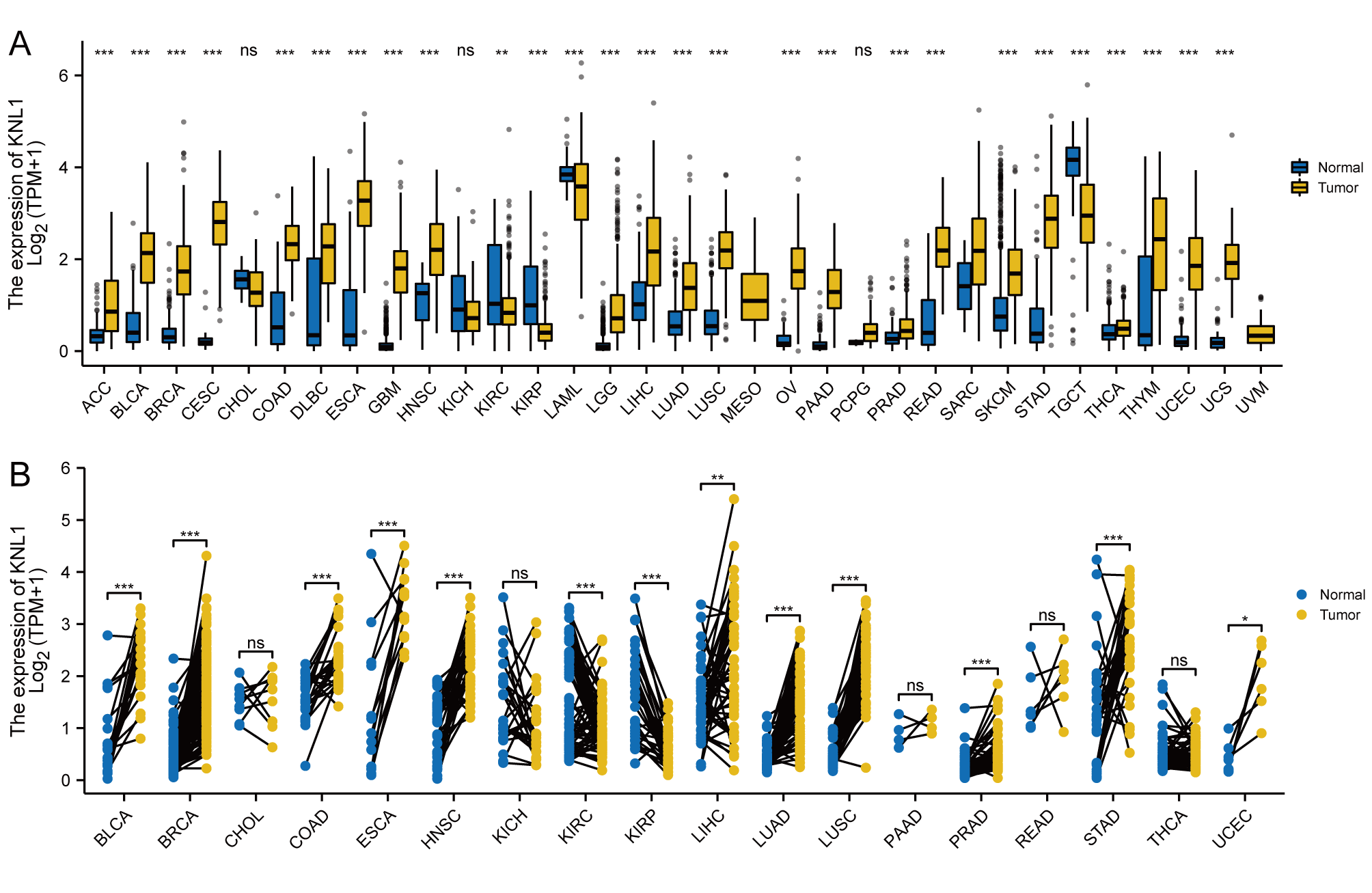

Supplement: Supplementary Figure 1 — Differential expression analysis results of KNL1 in pancancer patients. (A) Results of differential analysis of KNL1 expression in 33 tumors based on TCGA database data. (B) Pancancer analysis of paired samples based on data from the TCGA database. [file Image_1.tif]

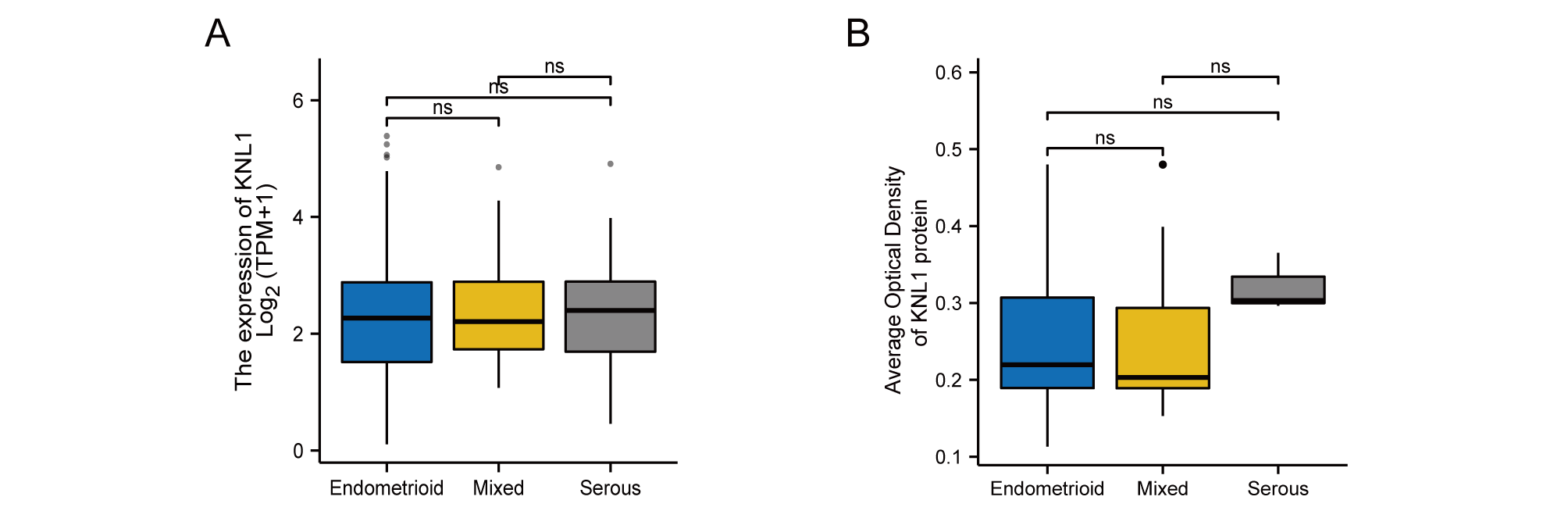

Supplement: Supplementary Figure 2 — Expression of KNL1 in UCEC patients with different histological types. (A) Box diagram of KNL1 expression obtained from RNA-seq data in the TCGA database. (B) Box plot of KNL1 expression using immunohistochemical analysis of 108 clinical samples. [file Image_2.tif]

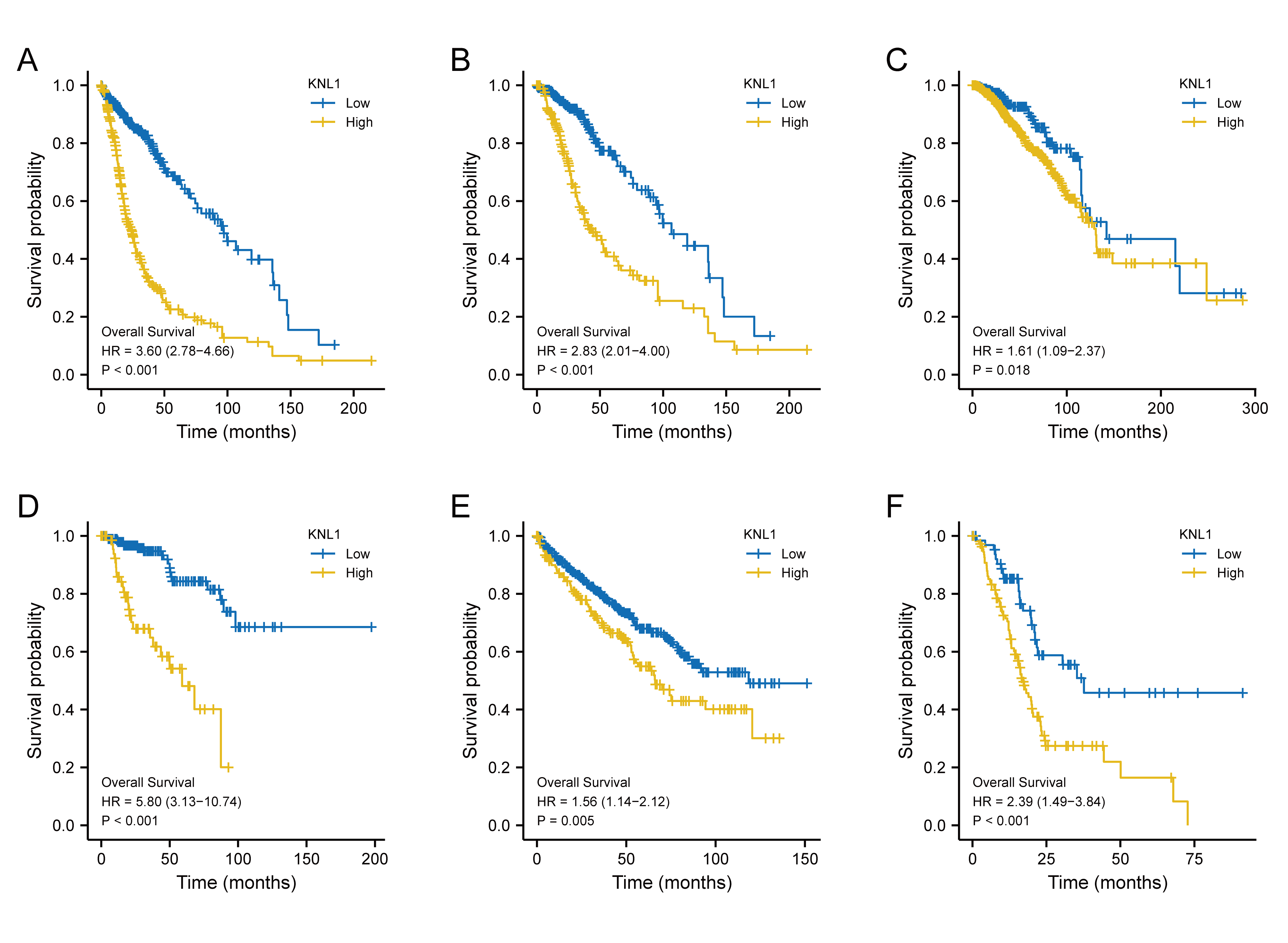

Supplement: Supplementary Figure 3 — KM overall survival curves stratified by KNL1 expression in different tumors. (A) KM survival curve of GBMLGG, (B) KM survival curve of LGG, (C) KM survival curve of BRCA, (D) KM survival curve of KIRP, (E) KM survival curve of KIRC, and (F) KM survival curve of PAAD. [file Image_3.tif]
