# Supplementary material for: KNL1 is a prognostic and diagnostic biomarker related to immune infiltration in patients with uterine corpus endometrial carcinoma
Source: Front Oncol. 2023 Jan 27;13:1090779. doi: 10.3389/fonc.2023.1090779 (PMC9913269; doi:10.3389/fonc.2023.1090779)
Supplement: Supplementary file 4 [file Table_1.docx]

| Category | No. Paired samples | | No. Non-paired samples | |
| --- | --- | --- | --- | --- |
|  | **Adjacent** | **Tumor** | **Normal** | **Tumor** |
| ACC |  |  | 128 |  |
| BLCA | 19 | 19 | 28 | 407 |
| BRCA | 112 | 112 | 292 | 1099 |
| CESC |  |  | 13 | 306 |
| CHOL | 9 | 9 | 9 | 36 |
| COAD | 26 | 26 | 349 | 290 |
| DLBC |  |  | 444 | 47 |
| ESCA | 13 | 13 | 666 | 182 |
| GBM |  |  | 1157 | 166 |
| HNSC | 43 | 43 | 44 | 520 |
| KICH | 25 | 25 | 53 | 66 |
| KIRC | 72 | 72 | 100 | 531 |
| KIRP | 32 | 32 | 60 | 289 |
| LAML |  |  | 70 | 173 |
| LGG |  |  | 1152 | 523 |
| LIHC | 50 | 50 |  | 371 |
| LUAD | 58 | 58 | 347 | 515 |
| LUSC | 50 | 50 | 338 | 498 |
| MESO |  |  |  | 87 |
| OV |  |  | 88 | 427 |
| PAAD | 4 | 4 | 171 | 179 |
| PCPG |  |  | 3 | 182 |
| PRAD | 52 | 52 | 152 | 496 |
| READ | 6 | 6 | 318 | 93 |
| SARC |  |  | 2 | 262 |
| SKCM |  |  | 813 | 469 |
| STAD | 33 | 33 |  | 414 |
| TGCT |  |  | 165 | 154 |
| THCA | 59 | 59 | 338 | 512 |
| THYM |  |  | 446 | 119 |
| UCEC | 7 | 7 | 101 | 181 |
| UCS |  |  | 78 | 57 |
| UVM |  |  |  | 79 |

**Supplementary Table 1. Sample size included in the pan-cancer analysis of paired and unpaired samples**
