# Supplementary material for: KNL1 is a prognostic and diagnostic biomarker related to immune infiltration in patients with uterine corpus endometrial carcinoma
Source: Front Oncol. 2023 Jan 27;13:1090779. doi: 10.3389/fonc.2023.1090779 (PMC9913269; doi:10.3389/fonc.2023.1090779)
Supplement: Supplementary file 5 [file Table_2.docx]

**Supplementary Table 2. GO and KEGG analysis**

| Ontology | ID | Description | GeneRatio | BgRatio | pvalue | p.adjust | qvalue |
| --- | --- | --- | --- | --- | --- | --- | --- |
| BP^a^ | GO:0002526 | acute inflammatory response | 32/709 | 220/18670 | 6.66e-11 | 9.41e-08 | 8.74e-08 |
| BP | GO:0006959 | humoral immune response | 37/709 | 356/18670 | 3.13e-08 | 1.66e-05 | 1.54e-05 |
| MF^a^ | GO:0001664 | G protein-coupled receptor binding | 27/697 | 280/17697 | 1.76e-05 | 8.30e-04 | 7.50e-04 |
| BP | GO:0042445 | hormone metabolic process | 20/709 | 232/18670 | 5.71e-04 | 0.027 | 0.025 |
| CC^a^ | GO:0045095 | keratin filament | 13/765 | 95/19717 | 7.56e-05 | 0.002 | 0.002 |
| MF | GO:0004867 | serine-type endopeptidase inhibitor activity | 12/697 | 94/17697 | 3.14e-04 | 0.009 | 0.008 |
| MF | GO:0005125 | cytokine activity | 18/697 | 220/17697 | 0.003 | 0.050 | 0.045 |
| MF | GO:0005179 | hormone activity | 18/697 | 122/17697 | 1.35e-06 | 7.42e-05 | 6.71e-05 |
| MF | GO:0043027 | cysteine-type endopeptidase inhibitor activity involved in apoptotic process | 5/697 | 25/17697 | 0.003 | 0.047 | 0.043 |
| CC | GO:0016342 | catenin complex | 6/765 | 29/19717 | 7.39e-04 | 0.016 | 0.014 |
| CC | GO:0005871 | kinesin complex | 8/765 | 55/19717 | 0.001 | 0.022 | 0.020 |
| BP | GO:0070192 | chromosome organization involved in meiotic cell cycle | 12/709 | 65/18670 | 5.20e-06 | 9.58e-04 | 8.90e-04 |
| CC | GO:0072686 | mitotic spindle | 13/765 | 109/19717 | 3.08e-04 | 0.007 | 0.006 |
| BP | GO:1903046 | meiotic cell cycle process | 20/709 | 188/18670 | 3.29e-05 | 0.003 | 0.003 |
| CC | GO:0000793 | condensed chromosome | 18/765 | 223/19717 | 0.003 | 0.042 | 0.038 |
| KEGG | hsa04080 | Neuroactive ligand-receptor interaction | 37/290 | 341/8076 | 1.15e-09 | 3.00e-07 | 2.86e-07 |
| KEGG | hsa04610 | Complement and coagulation cascades | 15/290 | 85/8076 | 2.76e-07 | 3.62e-05 | 3.45e-05 |
| KEGG | hsa00140 | Steroid hormone biosynthesis | 11/290 | 61/8076 | 8.93e-06 | 7.80e-04 | 7.42e-04 |
| KEGG | hsa00980 | Metabolism of xenobiotics by cytochrome P450 | 12/290 | 77/8076 | 1.66e-05 | 0.001 | 0.001 |
| KEGG | hsa05204 | Chemical carcinogenesis | 12/290 | 82/8076 | 3.20e-05 | 0.002 | 0.002 |
| KEGG | hsa00982 | Drug metabolism - cytochrome P450 | 10/290 | 71/8076 | 2.02e-04 | 0.009 | 0.008 |
| KEGG | hsa04976 | Bile secretion | 11/290 | 90/8076 | 3.52e-04 | 0.013 | 0.013 |
| KEGG | hsa00053 | Ascorbate and aldarate metabolism | 6/290 | 30/8076 | 5.83e-04 | 0.019 | 0.018 |
| KEGG | hsa04742 | Taste transduction | 10/290 | 86/8076 | 9.66e-04 | 0.028 | 0.027 |
| KEGG | hsa00040 | Pentose and glucuronate interconversions | 6/290 | 34/8076 | 0.001 | 0.031 | 0.029 |
| KEGG | hsa04970 | Salivary secretion | 10/290 | 93/8076 | 0.002 | 0.042 | 0.040 |

^a^ CC, Cellular Component; BP, Biological Process; MF, Molecular Function.
