# Supplementary material for: KNL1 is a prognostic and diagnostic biomarker related to immune infiltration in patients with uterine corpus endometrial carcinoma
Source: Front Oncol. 2023 Jan 27;13:1090779. doi: 10.3389/fonc.2023.1090779 (PMC9913269; doi:10.3389/fonc.2023.1090779)
Supplement: Supplementary file 6 [file Table_3.docx]

**Supplementary Table 3. Baseline datasheet**

| Characteristic | Low expression of KNL1 | High expression of KNL1 | p |
| --- | --- | --- | --- |
| n | 276 | 276 |  |
| Clinical stage, n (%) |  |  | 0.341 |
| Stage I | 181 (32.8%) | 161 (29.2%) |  |
| Stage II | 24 (4.3%) | 27 (4.9%) |  |
| Stage III | 57 (10.3%) | 73 (13.2%) |  |
| Stage IV | 14 (2.5%) | 15 (2.7%) |  |
| Primary therapy outcome, n (%)^a,b^ |  |  | 0.082 |
| PD | 11 (2.3%) | 9 (1.9%) |  |
| SD | 2 (0.4%) | 4 (0.8%) |  |
| PR | 2 (0.4%) | 10 (2.1%) |  |
| CR | 228 (47.5%) | 214 (44.6%) |  |
| Race, n (%)^a^ |  |  | 0.981 |
| Asian | 10 (2%) | 10 (2%) |  |
| Black or African American | 53 (10.5%) | 55 (10.8%) |  |
| White | 190 (37.5%) | 189 (37.3%) |  |
| Age, n (%)^a^ |  |  | 0.904 |
| <=60 | 104 (18.9%) | 102 (18.6%) |  |
| >60 | 170 (31%) | 173 (31.5%) |  |
| Weight, n (%)^a^ |  |  | 0.193 |
| <=80 | 114 (21.6%) | 129 (24.4%) |  |
| >80 | 151 (28.6%) | 134 (25.4%) |  |
| Height, n (%)^a^ |  |  | 0.174 |
| <=160 | 115 (22%) | 132 (25.2%) |  |
| >160 | 146 (27.9%) | 130 (24.9%) |  |
| BMI, n (%)^a^ |  |  | 0.842 |
| <=30 | 105 (20.2%) | 107 (20.6%) |  |
| >30 | 156 (30.1%) | 151 (29.1%) |  |
| Histological type, n (%) |  |  | 0.671 |
| Endometrioid | 208 (37.7%) | 202 (36.6%) |  |
| Mixed | 13 (2.4%) | 11 (2%) |  |
| Serous | 55 (10%) | 63 (11.4%) |  |
| Residual tumor, n (%)^a,c^ |  |  | 0.677 |
| R0 | 193 (46.7%) | 182 (44.1%) |  |
| R1 | 11 (2.7%) | 11 (2.7%) |  |
| R2 | 10 (2.4%) | 6 (1.5%) |  |
| Histologic grade, n (%)^a^ |  |  | < 0.001 |
| G1 | 72 (13.3%) | 26 (4.8%) |  |
| G2 | 71 (13.1%) | 49 (9.1%) |  |
| G3 | 128 (23.7%) | 195 (36%) |  |
| Tumor invasion(%), n (%)^a^ |  |  | 0.971 |
| <50 | 137 (28.9%) | 122 (25.7%) |  |
| >=50 | 115 (24.3%) | 100 (21.1%) |  |
| Menopause status, n (%)^a^ |  |  | 0.614 |
| Pre | 16 (3.2%) | 19 (3.8%) |  |
| Peri | 7 (1.4%) | 10 (2%) |  |
| Post | 232 (45.8%) | 222 (43.9%) |  |
| Hormones therapy, n (%)^a^ |  |  | 0.786 |
| No | 148 (43%) | 149 (43.3%) |  |
| Yes | 25 (7.3%) | 22 (6.4%) |  |
| Diabetes, n (%)^a^ |  |  | 0.436 |
| No | 170 (37.7%) | 158 (35%) |  |
| Yes | 58 (12.9%) | 65 (14.4%) |  |
| Radiation therapy, n (%)^a^ |  |  | 0.106 |
| No | 149 (28.3%) | 130 (24.7%) |  |
| Yes | 114 (21.6%) | 134 (25.4%) |  |
| Surgical approach, n (%)^a^ |  |  | 0.559 |
| Minimally Invasive | 101 (19.1%) | 107 (20.2%) |  |
| open | 166 (31.3%) | 156 (29.4%) |  |
| Age, median (IQR) | 64 (57, 72.75) | 64 (57, 70) | 0.778 |

^a^ Data incomplete as some record data were lost.

^b^ PD, Progressive Disease; SD, Stable Disease; PR, Partial Response; CR, Complete Response.

^c^ R0, no residual tumor; R1, microscopic residual tumor; R2, macroscopic residual tumor.
