# Supplementary material for: KNL1 is a prognostic and diagnostic biomarker related to immune infiltration in patients with uterine corpus endometrial carcinoma
Source: Front Oncol. 2023 Jan 27;13:1090779. doi: 10.3389/fonc.2023.1090779 (PMC9913269; doi:10.3389/fonc.2023.1090779)
Supplement: Supplementary file 7 [file Table_4.docx]

**Supplementary Table 4. Association between the expression of KNL1 and clinical features of patients with UCEC.**

| Characteristic | Low expression of KNL1 | High expression of KNL1 | p |
| --- | --- | --- | --- |
| n | 55 | 53 |  |
| Tumor invasion(%), n (%) |  |  | 0.027 |
| <50 | 44 (40.7%) | 31 (28.7%) |  |
| >=50 | 11 (10.2%) | 22 (20.4%) |  |
| Histological type, n (%) |  |  | 0.191 |
| Endometrioid | 47 (43.5%) | 45 (41.7%) |  |
| Mixed | 8 (7.4%) | 5 (4.6%) |  |
| Serous | 0 (0%) | 3 (2.8%) |  |
| Histologic grade, n (%)^b^ |  |  | < 0.001 |
| G1 | 32 (30.5%) | 8 (7.6%) |  |
| G2 | 17 (16.2%) | 38 (36.2%) |  |
| G3 | 3 (2.9%) | 7 (6.7%) |  |
| Lymphatic metastasis, n (%)^b^ |  |  | 0.554 |
| No | 35 (40.7%) | 37 (43%) |  |
| Yes | 5 (5.8%) | 9 (10.5%) |  |
| FIGO stage, n (%)^a^ |  |  | 0.002 |
| Stage I | 46 (42.6%) | 28 (25.9%) |  |
| Stage II | 2 (1.9%) | 11 (10.2%) |  |
| Stage III | 5 (4.6%) | 12 (11.1%) |  |
| Stage IV | 2 (1.9%) | 2 (1.9%) |  |
| Age, n (%) |  |  | 0.556 |
| <55 | 28 (25.9%) | 23 (21.3%) |  |
| >=55 | 27 (25%) | 30 (27.8%) |  |
| Radiation therapy, n (%) |  |  | 0.156 |
| No | 44 (40.7%) | 35 (32.4%) |  |
| Yes | 11 (10.2%) | 18 (16.7%) |  |
| Menopause status, n (%) |  |  | 0.731 |
| No | 18 (16.7%) | 20 (18.5%) |  |
| Yes | 37 (34.3%) | 33 (30.6%) |  |
| Diabetes, n (%) |  |  | 0.168 |
| No | 49 (45.4%) | 41 (38%) |  |
| Yes | 6 (5.6%) | 12 (11.1%) |  |
| Ki67, median (IQR) | 40 (30, 70) | 70 (50, 75) | < 0.001 |

^a^ FIGO, International Federation of Gynecology and Obstetric

^b^ Data incomplete as some record data were lost.
